# Challenges of Egg, Sperm, and Embryo Donation from the Perspective of Iranian Experts: A Qualitative Study

**DOI:** 10.34172/aim.33938

**Published:** 2025-06-01

**Authors:** Mostafa Saeedinia, Shirin Shahbazi Sighaldeh, Kobra Joodaki, Sedigheh Hantoushzadeh, Fedyeh Haghollahi, Saghar Salehpour, Majid Zaki-Dizaji, Nikzad Isazadeh, Zohreh Heidary

**Affiliations:** ^1^Student Research Committee, School of Medicine, Shahid Beheshti University of Medical Sciences, Tehran, Iran; ^2^Midwifery and Reproductive Health Department, School of Nursing and Midwifery, Tehran Universities of Medical Sciences, Tehran, Iran; ^3^Nursing and Midwifery Care Research Center, Tehran University of Medical Sciences, Tehran, Iran; ^4^National Association of Iranian Obstetricians and Gynecologists (NAIGO), Tehran, Iran; ^5^Vali-E-Asr Reproductive Health Research Center, Family Health Research Institute, Tehran University of Medical Sciences, Tehran, Iran; ^6^Preventative Gynecology Research Center, Shahid Beheshti University of Medical Sciences, Tehran, Iran; ^7^Human Genetics Research Center, Baqiyatallah University of Medical Sciences, Tehran, Iran; ^8^Research Center of Quran, Hadith and Medicine, Tehran University of Medical Sciences, Tehran, Iran

**Keywords:** Assisted reproductive methods, Guideline, Infertility treatment, Qualitative approach, Registry

## Abstract

**Background:**

Assisted reproductive technologies (ARTs) have been implemented in Iran for nearly thirty years. However, except for embryo donation, none of the other cases, including embryo and sperm donation have not yet been legalized through parliamentary resolutions. This study aims to explore the challenges of these three types of donations from the perspective of fertility experts.

**Methods:**

This qualitative exploratory research was conducted in Tehran in 2024. Participants consisted of 12 specialists with experience in infertility treatment or other fields related to embryo, sperm and egg donation, who were selected through purposive sampling. Data were collected via focus group discussions and analyzed based on conventional content analysis using MAXQDA 20.

**Results:**

Data were classified into 12 categories. Eight categories describe the challenges and problems of egg, sperm, and embryo donation and 4 categories describe the solutions recommended by experts.

**Conclusion:**

According to the study findings, sperm donation faces the most legal and medical challenges compared to egg and embryo donation. Designing a comprehensive national protocol could be the best solution proposed to Iranian health policymakers. Conducting more qualitative and mixed methods studies related to sperm, egg, and embryo donation could confirm and strengthen the findings of this study.

## Introduction

 Infertility is a common problem for couples, with psychological, economic, demographic, and medical considerations. The demand for infertility services has increased significantly. Unfortunately, owing to the prevalence of infertility in Iran (10%–15%) and, globally, the changing living conditions, this rate is constantly increasing worldwide and in Iran.^1-4^

 In general, 50% of infertility cases are caused by a purely female factor. A total of 20%–30% of the problem is the male factor, and the remaining 20%–30% is due to a combination of both female and male factors.^5^

 The World Health Organization (WHO) has recognized that infertility should be considered an all-purpose disease that impairs the health and well-being of its sufferers.^6^ Currently, ART is the most effective method for treating infertility. A variety of fertility aids are used to help these couples have children. These aids are commonly used in most countries around the world but are still subject to religious, social, moral, political, and medical debates. ART depends on various factors, including social structure, political choices, moral issues, and religious beliefs.^7^ This supposedly makes it impossible to establish a standard regulation in different countries, especially in controversial cases such as donation of gametes and embryos.^8^

 Assisted reproductive technologies (ARTs) have been implemented in Iran for nearly thirty years. All their forms, including egg donation, sperm donation, and embryo donation, are carried out in Iranian IVF centers.^9-10^ Nevertheless, except for embryo donation, none of the other cases, including egg and sperm donation, although approved, have been legalized yet through parliamentary resolutions.

 The use of sperm, egg, and embryo donation has grown significantly in recent decades,^11^ offering individuals and couples struggling to conceive a chance to start a family. Organizations like the Food and Drug Administration (FDA), the Centers for Disease Control and Prevention (CDC), and American Society for Reproductive Medicine (ASRM) have set guidelines for screening donors and recipients.^12^

 In other words, the law on embryo donation passed by the parliament is not comprehensive. This method cannot solve the problems created, especially in the field of egg and sperm donation.^8^

 In Iran, the Guardian Council, in its meeting on September 27, 2002, regarding the resolution of September 5, 2018, by the Islamic Council of Iran, did not consider the principle of embryo transfer illegal and unconstitutional. However, experts believe that the bill is not comprehensive and that no regulations have been established for other cases of assisted reproduction that are considered illegal methods in the law. Currently, for example, sperm donation is not legal or regulated egg donation. If these methods of assisted reproduction can be examined from the principles of heredity and embarrassment approved by jurists, the associated challenges can be solved as much as possible.^13^ ARTs have been implemented in Iran for nearly thirty years. However, except for embryo donation, none of the other cases, including embryo and sperm donation have yet not been legalized through parliamentary resolutions. This study aims to explore the challenges of these three types of donations from the perspective of fertility experts.

 Notably, owing to the need to find a way to have children for infertile couples and, on the other hand, legislation on these issues under the existing laws as well as taking into account religious and socio-cultural concerns, it is necessary to discuss these issues separately and carefully in the presence of relevant professionals. This study aims to outline the perspectives of experts on the legal and ethical issues surrounding infertility treatments, including embryo, egg, and sperm donation, and to propose solutions. The findings from these expert discussions can significantly assist health ministries and medical education authorities in their decision-making processes in this field.

## Materials and Methods

 In this qualitative study, after informed consent was obtained from the participants and the research objectives were stated by the researcher, group interviews were conducted with 12 related experts in October 2024 at the reproductive health research center affiliated with Tehran University of Medical Sciences. Specialists came from a variety of disciplines, including gynecology and obstetrics, reproductive health, medical genetics, law, jurisprudence, and medical ethics. During the sessions, the leaders of the focused groups, who were the principal researchers, encouraged participants to talk to each other openly and freely regarding the main research questions (Box 1). The interviews, which were based on the interview guide, started with general questions about infertility assistance treatments in Iran and then focused on challenges related to embryo, egg, and sperm donation. The voices of the participants were recorded, and their key points were noted. After the meeting, the recordings were transcribed verbatim and then analyzed by two of the project’s researchers.

**Box 1. Final Draft of Research Questions in the Interview Guide**

1. Please share your experience in assisted reproductive therapy.

2. Please tell us about egg donation, expressing your knowledge, perspective, or work experience in this field.

- Based on your work experience, what are the challenges of egg donation in Iran?

- Please talk more about the legal and ethical challenges.

- What do you think about the circumstances or backgrounds that lead to these challenges?

- What do you think about the way to solve or prevent these challenges?

- Is there anything more you would like to add?

3. Please tell us about sperm donation, expressing your knowledge, perspective, or work experience in this field.

- Based on your work experience, what are the challenges of sperm donation in Iran?

- Please talk more about the legal and ethical challenges.

- What do you think about the circumstances or backgrounds that lead to these challenges?

- What do you think about the way to solve or prevent these challenges?

- Is there anything more you would like to add?

4. Please tell us about embryo donation, expressing your knowledge, perspective, or work experience in this field.

- Based on your work experience, what are the challenges of embryo donation?

- Please talk more about the legal and ethical challenges.

- What do you think about the circumstances or backgrounds that lead to these challenges?

- What do you think about the way to solve or prevent these challenges?

- Is there anything more you would like to add?

Since in qualitative studies, obtaining rich data through in-depth interviews is more important than the number of participants, an attempt was made to invite key experts to conduct interviews, and during a session that lasted about 4 hours, their experiences were fully extracted to answer the research questions. Since the interviewees were among the key informants and the most famous experts in the fields related to embryo, sperm and egg donation, and after analyzing the data, there was no need to obtain more information because the researcher reached a data saturation by asking numerous questions and obtaining appropriate answers, and therefore sampling was stopped after conducting one group discussion with 12 participants.

 The group discussion began with asking an open-ended question by the Principal Investigator (PI). All participants were asked to respond to each question individually. Participants were also asked to provide any comments they had about the other participants’ discussions so that each question raised could be fully described and explained. A facilitator managed the time each expert spent answering each question to ensure that there was enough time for all participants. The time each participant spent speaking was recorded and summarized to ensure that sufficient and fair time was allocated to each participant. Since all participants were enthusiastic about sharing their experiences and perspectives and the facilitator managed the time well, at the end of the session, all participants stated that they did not need more time to present their opinions and that the session had been sufficient to convey their experiences and perspectives.

 After the session, data analysis and review of trustworthiness was done by the PI and one of the colleagues proficient in qualitative research. Data analysis was performed through the MAXQDA 20 software to manage and categorize the data. Since data analysis was conducted using a conventional content analysis method, the coding process was completely emergent and no predetermined categories were used. To check the researchers’ agreement on the selected codes, the PI sent the initial codes frame and the interview transcripts to all members of the research team and made the necessary changes after obtaining their opinions. Then, the final codes were compared with each other and similar codes were placed together in a category. Finally, appropriate labels were selected to classify them in 12 categories. The selection of categories was also made with the agreement of all members of the research team. It is worth noting that during the coding process, no contradictory findings were observed that would have prompted the need for additional interviews.

 Data trustworthiness was further assessed by providing extracted codes and categories to the experts participating in the study, achieving a deep understanding of the analyzed data, providing participants’ demographics, and clearly describing the research steps.

## Results

 The participants’ ages ranged from 40 to 60, and their work experience was between 8 and 30 years. Table 1 shows the other demographic characteristics of the participants.

**Table 1:** Characteristics of Study Participants

**Gender**		**Field of study**	**Education**	**Occupation**
Participant No. 1	Female	Gynecology and obstetrics	Infertility fellowship	Infertility specialist
Participant No. 2	Female	Medical ethics	MD	Medical ethicist
Participant No. 3	Male	Islamic teachings	PhD	Head of the Center for Research in Quranic, Hadith, and Medical Sciences
Participant No. 4	Female	Gynecology and obstetrics	Prenatal fellowship	Head of the Institute
Participant No. 5	Female	Maternal and child health specialist	Maternal and child health specialist	Head of the research center
Participant No. 6	Male	Medical genetics	PhD	University teacher
Participant No. 7	Female	Medical genetics	PhD	University teacher
Participant No. 8	Female	Molecular genetics	PhD	Research expert
Participant No. 9	Female	Medical ethics	PhD	University teacher
Participant No. 10	Female	Obstetrics	MSc	Director of research
Participant No. 11	Female	Reproductive health	PhD	University teacher
Participant No. 12	Male	Private law	PhD	Judge of the court

 Although assisted reproduction methods have solved the problem of infertility in many infertile couples and given new hope to childless families, they face challenges and consequences that require discovery and attention. According to one of the participants in the study, *“The important issue in medical fertility assistance is to solve the problem of infertility and having children in a family, and then it is the child’s rights arising from the process of medical fertility assistance”.* However, all aspects of the process of childbearing through assisted reproduction methods should be clear to both planners and doctors and parents and donors so that all decisions and choices are made on the basis of full awareness and reality. In this way, the possibility of negative consequences is minimized, and the pleasure of being parents is not diminished by solvable concerns. The following is a description of the qualitative data of the study, which are classified into 12 categories. These categories describe the challenges and problems of egg, sperm, and embryo donation (Table 2) and the proposed solutions (Table 3) as follows:

**Table 2:** Categories of Qualitative Data: Challenges and Problems Related to Egg, Sperm, and Embryo Donation

**Category**	**Merged or Highest Frequency Codes**
Outcomes and consequences of assisted reproductive treatment (ART)	Autism
	Cancer
	Genetic diseases
	Possible marriage of siblings
	Embryo anomalies
	Return of the donated child
	Genetic transmission of addiction
	Disruption of the gender ratio
	Death or psychological trauma of the donor
	Deterioration of the country's international image
	An increase in pregnancy complications such as preeclampsia and neonatal complications such as prematurity in cases of embryo donation
Religious challenges regarding assisted reproduction	Lack of global agreement on embryo, sperm, and egg donation
	Disagreement between Shiites and Sunnis regarding the donation of embryos, sperm, and eggs
The big challenge: sperm donation	Repeated sperm donation by any donor (third parent)
	The risk of inbreeding due to the possible marriage of offspring resulting from the donation of the third parent
	The possibility of transmission of genetic and infectious diseases through sperm
	The necessity of keeping the information related to the donation confidential and not disclosing it to anyone other than the person receiving the donation
Donor-related challenge: the mental health challenge	The possibility of serious mental disorders in the donor and their transmission to the fetus
The challenge of inheritance, lineage, and identity	Seeking to identify biological parents for inheritance and financial support.
	The desire to identify biological parents to understand his true identity
	The epidemic of children's doubts about their genetic origin
Lack of national law and protocol	Lack of law and protocol regarding permitted activities and how to perform them in infertility centers
	Parallel work of infertility centers regarding the method of donation due to not having the same protocol
The brokerage market for egg and sperm donation	The business of donating sperm and eggs due to lack of supervision by the Ministry of Health
	The unnecessary use of assisted reproductive methods as a result of indiscriminate expansion of infertility centers.
General challenges of infertility treatment	Lack of a unified protocol among infertility centers.
	Absence of a registry system
	Lack of necessary facilities in government centers to introduce the donor to the patient.
	Absence of jurisprudence and moral advisor
	Charging excessive fees from liaisons while failing to monitor private center operations.

**Table 3:** Categories Derived from Qualitative Data: Expert Suggestions for Addressing Donation-Related Challenges

**Categories**	**Merged or Highest Frequency Codes**
Central registry	Preventing inbreeding among donor offspring
	Tracking the number of sperm donations from each man
	Avoiding secrecy and incestuous marriages
	Identifying the genetic origins of donor children for necessary bone marrow transplants
	Ensuring that donor children can inherit from a third parent
	The right of donor children to know their genetic origins
	Maintaining data confidentiality
Medical and legal advice before donation	Conducting genetic counseling specific to the region
	Reviewing histories of incurable infectious diseases and mental disorders.
	Assessing the drug use history of both the donor and recipient.
	Considering the donor's financial status.
	Reviewing ethical competency
	Ensuring that the donor has no mental disorders and is a citizen of the Islamic Republic.
	The necessity to highlight the potential for serious future diseases in children.
	A need to state the possibility of claiming the child's inheritance from the donor.
	Obtaining clear and comprehensive informed consent.
	Collecting cord blood samples.
Drafting and communicating the amendment bill and presenting the national protocol	The necessity of establishing a unified legal article for all infertility centers to follow.
	The need to clarify the issue of attribution of lineage in the law
	The protocol must specify the age, conditions, and individuals responsible for informing about the donation.
	The protocol should specify the conditions of sperm, embryo, and egg donation in all centers.
Research and support from laboratories, government officials, and scientific and religious leaders	Conducting qualitative and quantitative studies to identify issues in infertility centers.
	Consultation with scientific, jurisprudential, and legal experts
	Utilizing studies from the Judiciary Research Center and the Guardian Council on donations.
	Referring the issue to the religious leader

###  Challenges and Problems Related to Egg, Sperm, and Embryo Donation

####  Outcomes and Consequences of Assisted Reproductive Treatment (ART)

 Based on the participants’ experience, there is a possibility of complications such as autism or cancer in children from assisted reproduction methods. The occurrence of a tsunami of genetic diseases due to inbreeding is another consequence that experts emphasized: *“Diseases such as thalassemia, tuberculosis, cystic fibrosis, etc., are the result of inbreeding. While we have many ethnicities and family marriages, we will seriously suffer from these diseases.”*

 One of the consequences of ART is the possibility of sibling marriage and an increased likelihood of genetic disease: “These people are more likely to develop cancer and embryo anomalies.” Notably, these diseases are rare complications of ART treatment, but given the high complication rate of these diseases, researchers emphasized them.

 The experts also suggested the possibility of genetic transmission of addiction as another consequence of donation: *“On the other hand, addiction and a history of substance abuse are important. It has now been proven that addiction has a genetic background. These must be controlled.”*

 The use of assisted reproductive methods raises ethical concerns, particularly regarding the choice of a child’s gender or appearance. Many are questioning the morality behind practices such as gender determination, which some consider unnecessary. Infertility treatment centers, especially in wealthier areas, have begun to prioritize personal preferences in their services, asking clients to consider what gender their child should be or what eye color they desire.

 Moreover, the legal implications of these practices cannot be overlooked. There have been instances where donors, after having their children years later, have sought to reclaim a child conceived through donation. Such lawsuits reflect the complex and often troubling intersection of donor rights and parental claims, urging society to carefully examine the ramifications of these reproductive technologies.

 The health of donors is also at risk. Donors often come from economically pressured backgrounds, leading them to accept the risks associated with donation. For example, *drugs used for egg donation can cause serious complications; where for example, an 18-year-old girl died from these complications*. Additionally, mental health issues may arise if donors discover hidden diseases during pre-donation screenings. *Studies indicate that if a person tests positive for a genetic disease or HIV, it can have a devastating impact on their life*. *Lack of national laws and protocols for donations also tarnishes the country’s international reputation*. *Without comprehensive legislation, practices can vary widely, and some organizations may exploit this lack of regulation, damaging our global image*. Furthermore, this absence of regulations can lead to an increase in pregnancy complications for mothers, such as preeclampsia and premature births, which in turn increases the risk of complications for infants. *One expert noted that in embryo donation, 100% of the genes are foreign, which can trigger reactions in the mother’s immune system. This can result in a greater incidence of issues such as preeclampsia, premature birth, embryo diseases, high blood pressure, and developmental delays for the baby.*

####  Religious Challenges Regarding Assisted Reproduction

 Experts note that worldwide, religious leaders—both in Islamic and non-Islamic countries—have not reached a consensus on issues related to embryo donation, cloning, or surrogacy. In many Islamic countries, these practices are either rejected outright or considered acceptable only under specific conditions, such as the establishment of a central registry. *For example, at a conference attended by representatives from countries such as Iraq, Syria, and Palestine, there was a strong sentiment against procedures such as IVF unless a registry was in place*. The participants argued that infertility treatments should not proceed without this oversight.

*Moreover, experts highlighted the unique religious challenges faced in Iran. Current laws do not adequately address the donation of eggs or sperm between individuals of different religious backgrounds. This raises complex questions regarding donations between Shia and Sunni Muslims, between Iranian and foreign individuals, and between Muslims and non-Muslims (such as Zoroastrians, Christians, and Jews). Additionally, there are concerns about donations between members of the Sadat community and non-Sadat individuals. The existing legal framework does not provide clear guidance in any of these situations.*

 One of the lawyers stated that the embryo donation law had been approved by the Guardian Council. He emphasized that legal and Sharia issues related to donation should be addressed rather than questioned. *He argued, “If there are legal and Sharia challenges, instead of abandoning the principle and depriving people of the opportunity to adopt, we should work on resolving those issues through legal means.*” The expert further noted that if the religious challenges surrounding childbearing are not addressed, Iranian families may become disheartened. *Many people could end up going abroad at great expense for infertility treatments to have children. Moreover, those without sufficient financial resources might resort to unauthorized infertility treatment centers in the country and, overall, turn to “underground” facilities.*

 According to the religious scholars involved in the study, Sunnis completely oppose assisted reproduction methods. He emphasized that the existence of different jurisprudential views on donation necessitates further examination and discussion. He stated, “*The Sunni perspective on assisted reproductive methods is one of absolute prohibition. In contrast, Shiite jurists argue that individuals who cannot conceive naturally have the right to pursue medical fertility options. There are five to six jurisprudential views on this subject, each with its rationale, which should all be discussed and summarized in this plan.”*

####  The Big Challenge: Sperm Donation

 Experts consider sperm donation to be the most significant challenges in donor assistance. This is largely because women have a more limited capacity for donation than men. According to one expert, “*The challenge of embryo donation and sperm donation is much less. When you want to fertilize an egg, you need at least 6 weeks, and 8–10 eggs are eventually obtained. However, a young man can give sperm 3 times a day. The number of embryos resulting from these sperm cannot be calculated, and all this becomes a challenge.* “ *Another expert added that although sperm donation is generally seen as free of complications, it is vital to conduct thorough screenings for genetic and infectious diseases to protect everyone involved.*

 The participants discussed the challenges associated with sperm donation, particularly the issues surrounding confidentiality and the concept of a “third parent.” Since a man can produce and donate a significant amount of sperm during his fertility period, several children in society can be conceived from a single sperm donor. These children are referred to as “third parents,” and one donor can potentially become the biological parent to many offspring.

*To illustrate this, a normal man has a sperm count of approximately 50--60 million sperm per cubic centimeter (CC). In microinjection procedures, only one sperm is needed for each egg fertilization. Therefore, if a man provides a sample even once a day, the potential for numerous pregnancies is substantial.*

 One expert *emphasized that the challenge of maintaining secrecy is now more of a concern than a serious obstacle. With rapid advancements in technology and genetics, a person’s genetic identity can easily be revealed through saliva samples. Genetic clinics can analyze an individual’s HLA or DNA not only to diagnose diseases but also to establish familial links. This has led to a troubling erosion of confidentiality*. Another expert warned, “*What worries us most is the risk of sibling marriages and the consequences that may follow.”*

 When discussing confidentiality, one expert elaborated, stating that personal information should only be shared with the individual from whom it originated. This underscores the critical need to safeguard genetic information from unauthorized access. *Our conversation about confidentiality is not just about an individual’s right to their genetic data following diagnosis; it also involves the legal duty to protect that information for everyone’s sake. Breaching this confidentiality is a serious offense and can create significant challenges for both the individual and their family.*

####  Donor-Related Challenge: Mental Health Challenge

 The experts emphasized the importance of examining the mental health of donors. They noted, “*Alongside this, we face challenges related to both recipient and donor psychiatry. Do they have mental health issues that could lead to significant problems? Additionally, what about complications such as bipolar disorder, schizophrenia, and depression? For example, can a 20-year-old with schizophrenia and a wife who is depressed still be eligible to donate embryos*?”

####  The Challenge of Inheritance, Lineage, and Identity

 One of the lawyers discussed the inheritance rights of children resulting from various donation methods, stating, “*The issuance of the family court license, as per Article 4 in the decree of the will, grants a third of the property of each receiving spouse to the child or children involved.” However, a child born through this method might seek to identify their biological parents after realizing that their legal parents are not their biological ones. This could lead to questions about inheritance and financial resources. If this tendency becomes widespread, many children may begin to doubt their genetic origins and embark on a search for their biological identities. To prevent potential misuse of this situation, the legislature needs to provide a clear and competent solution within the law*.”

 A medical ethicist noted, “*In jurisprudence, there is a rule regarding the attachment of the child to the husband. The Center for Judicial Research has been examining this issue for about ten years, but they have not achieved satisfactory results in resolving the question of familial relationships.” Another pressing issue is how a child can ascertain the identity of their parents. Historical insights indicate that determining a child’s lineage may rely on genetic tests, which can reveal whether the child shares a biological connection with a parent, regardless of ethnic or racial background.*

####  Lack of National Law and Protocol

 Experts have highlighted the absence of clear standards, laws, and a national protocol regarding authorized activities in infertility centers as significant challenges in infertility treatment in Iran. These centers are currently engaged in donation and gender determination practices without having an approved protocol in place for these activities. As one expert noted, “*Donation centers should be identified and authorized by the Ministry of Health. Currently, some centers operate primarily on donations and perform gender determination, but they lack formal oversight.*” This gap in legislation has created a situation where donation practices can vary widely, allowing donations to occur without consistent guidelines, regardless of the donor’s age or gender preference. *Furthermore, lack of legislation has resulted in inconsistent practices across different centers*. *This leads to a “parallel system” for embryo donation, where various processes may be used, and many of these practices are not adequately regulated*. One of the key issues being raised is the need to address the deficiencies in current laws regarding how embryo donations are handled.

 A medical ethicist involved in the study described the issue of embryo donation as a long-standing problem that has persisted for 20 years. The most significant concern is the absence of a national protocol. *From the onset of embryo donation, there have been jurisprudential discussions, legal articles written, and debates raised at infertility congresses. The conclusion drawn is that the core issue is lack of a national protocol, which is a concern, shared by compassionate individuals in society. The complications and challenges that arise are not due to the absence of a registry but rather from lack of a protocol. While some of these issues might be addressed through a registry, the fundamental problems are rooted in the absence of a clear protocol. A geneticist participating in the study echoed this sentiment, stating, *“*The main problem we have is not having a protocol*.”

####  The Brokerage Market for Egg and Sperm Donation

 This is a concern regarding infertility treatment in Iran. Lawyers have highlighted the commercialization of these treatments, which should be utilized strictly under the law for couples whose infertility has been scientifically proven. *This is clearly stated in Article 1 and Paragraph 1 of Article 2 of the relevant legislation. The law permits embryo donation only to couples who have been verified to be infertile, either individually or jointly, after marriage and following medical evaluations that confirm their inability to conceive, supported by a valid medical certificate. However, owing to inadequate oversight from the Ministry of Health, these procedures are being conducted commercially. There are reports of individual brokering arrangements for donors and recipients. The number of infertility centers has increased, and there are even cases where newly married individuals, such as a man who has been married for only three months, are offered in vitro fertilization (IVF) services.* One of the participants added: “*The issue of embryo donation must have a guardian and will not be resolved through parallel work (Performing the same tasks by the same centers or performing repetitive tasks)... Donation centers must be designated and announced by the Ministry of Health and Treatment because currently, there are centers whose livelihood is provided through donation and gender determination. Therefore, when there are no laws and regulations for all cases, the issue of donation is carried out everywhere, at any age and with the desired gender.”*

####  General Challenges of Infertility Treatment

 Based on the experience of one participant, several issues regarding donations for infertility treatment have been identified. These include 1) the absence of a unified protocol among infertility centers; 2) lack of a registry for donors; 3) insufficient facilities in government centers to facilitate introductions between donors and patients; 4) the need for a jurisprudential and ethical advisor; 5) high costs associated with consultations and lack of oversight in the operations of private centers.

###  Expert Suggestions for Addressing Donation-Related Challenges

####  Central Registration

 One of the key strategies recommended by the experts is the establishment of a registry for embryo, egg, and sperm donations. This initiative is seen as an effective way to prevent potential issues related to cohabitation among children conceived through donation. The registry would allow for tracking the number of sperm donations made by an individual, ensuring that each donor can donate only a limited quantity. The experts highlighted the importance of having a trusted and legal entity manage the donation process, thereby preventing the potential for concurrent donations. They proposed that the registry should be managed by the Census Bureau, with oversight from the Ministry of Health. This system not only ensures confidentiality but also allows for the determination of the genetic origins of offspring when necessary, such as in cases requiring a bone marrow transplant. Experts underscore that awareness of one’s genetic origins is a fundamental human right.

 The registry also allows a child to benefit from the inheritance of a third parent if applicable. *One significant issue surrounding the registry is the confidentiality and treatment of the child in the future. A child born from donated genetics should have the right to know their genetic origins, especially if they require a bone marrow transplant. Knowing one’s genetic background is a fundamental human right. Additionally, the topics of inheritance and familial connections arise. These children may seek inheritance from their biological parents, but other heirs could dispute their claims, depriving them of their rightful inheritance*. *The registry aims to address potential genetic issues, including the risk of hereditary diseases. If genetic compatibility concerns are not managed, they could lead to significant public health challenges*. The primary purpose of the registry is to secure genetic origin information for the child’s treatment while maintaining strict confidentiality by national and international laws. *Therefore, it is essential to establish legal provisions that grant access to this information only in cases of illness and for treatment.*

####  Medical and Legal Advice Before Donation

 Before donating, *it is essential to receive appropriate medical and legal advice. This includes consultations with specialists, genetic counseling, and thorough background checks on the donor, with a focus on substance use, mental health, and financial stability*. *Pre-donation counseling should specifically address genetic factors, potential addiction issues, and psychiatric concerns. Importantly, the donor should not be dealing with addiction and should be financially secure, so they can make a meaningful contribution on behalf of the child receiving the donation.*

 The experts emphasized the importance of ensuring that neither donors nor applicant couples become overly dependent on one another. They highlighted the necessity of evaluating moral competence, ensuring that donors do not suffer from incurable diseases, and confirming that they hold the citizenship of the Islamic Republic. These factors are essential conditions for donation. One expert discussed *the need for pre-donation consultations, stating that the needed genetic consultations and tests should be clearly defined. For example, tests for infectious diseases such as hepatitis should be categorized as mandatory. Additionally, certain tests, such as those related to prevalent conditions in specific provinces—such as thalassemia—should be also made mandatory and included in the donation protocol*. *Donations from individuals with a history of highly prevalent diseases, such as autism, should not be accepted.*

 Providing informed consent and educating infertile couples about the long-term risks and complications of donation were emphasized during the pre-donation counseling sessions. One participant from the University’s infertility clinic mentioned that *while thorough counseling is provided to couples before donation—including necessary health checks and informed consent—the details in the consent forms are not very clear*. One participant noted, “*We conduct various assessments, including genetic and psychiatric counseling, addiction screening, and donor health evaluations. Patients are informed about the potential complications of IVF. However, the informed consent forms do not clearly outline important issues, such as the possibility of sibling marriages in the future. Research has shown that these marriages can lead to increased risks of cancer and embryo anomalies. It is also crucial to inform donors during counseling that the children resulting from donation may seek inheritance rights later*.”

* An expert acknowledged that, despite discussing potential complications, couples often choose assisted reproduction techniques because of cultural pressures to have children. After consulting with patients, the office counselors informed them about these complications. Notably, four out of ten patients sought IVF services elsewhere, influenced by cultural beliefs that shaped their decisions*.

 A participant recommended that children conceived through assisted reproductive methods have their umbilical cord blood stored for potential disease treatment, emphasizing the need for this topic in counseling and informed consent: “*Children born via donation should have their cord blood stored for future medical use.” It is essential to encourage families to store cord blood*.

####  The Necessity of Amending the Law and Providing a National Protocol for the Donation of Egg, Sperm, or Embryo)

 The experts emphasized that *the Ministry of Health and Medical Education must address the dual issues of work and the shortcomings in current donation laws by collaborating with relevant agencies to draft a bill for legal amendments. “A unified legal framework is essential for all centers to follow*. Resolving the issues that donors and recipients face will also address our broader challenges. *Genealogy is a critical concern highlighted by participants in this study regarding embryo donation laws, necessitating input from scholars, religious leaders, and lawyers.*

*If we can resolve the ‘attribution of genealogy’ for these children, it will help clarify other legal matters such as inheritance, custody, alimony, and privacy rights.”*

*The concept of lineage refers to heredity. Establishing an inheritance relationship between children conceived through medical fertility and their legal parents and the right to mutual inheritance is essential to understanding the child’s lineage. Jurisprudential discussions typically focus on issues of inheritance, lineage, and alimony, all of which relate to individual rights.*

 A medical ethics expert emphasized *the need for a national protocol regarding donation cases to help prevent corruption. They noted that established protocols are common practices in other countries. If a child is conceived using the sperm and egg of their legal parents, it is acceptable to disclose that assisted reproductive methods were employed. However, revealing the origins of donors can complicate the child’s understanding of their identity and biological heritage. Moreover, if a child requires a bone marrow transplant, knowing their genetic background is crucial for finding a suitable donor. Therefore, key considerations, including when and how to inform the child about their origins, should be included in the protocol. Recently, the Royan Institute has implemented specific internal practices, such as rejecting sperm donations and limiting embryo transfers to a maximum of two*.

####  The Necessity of Conducting Scientific and Jurisprudential Research on Donation

 The participants emphasized the importance of research as a solution to the challenges associated with donations. They stated, “We need to engage with jurisprudential studies, such as those from the Center for Jurisprudential Studies of the Judiciary and the Research Center of the Guardian Council. We should present our issues to them and seek their assistance.” Infertility specialists should also share their insights into the problems they are facing. Therefore, conducting a qualitative study involving these specialists is essential for developing a comprehensive proposal for this research project. By identifying specific issues and injuries, we can explore potential solutions within jurisprudential, legal, and regulatory frameworks. A proposal has been made to assess infertility treatment centers to understand existing problems. Field work will be conducted to gather and categorize these issues, helping us broaden our perspective and improve our approach to the subject.

 A participant emphasized the need to refer the donation issue to the leader: “*We should summarize our concerns and inform the religious leader about the potential for sibling marriages to prevent complications related to inbreeding.”*

## Discussion

 Despite advancements in ARTs such as sperm, egg, and embryo donation, ethical issues remain a topic of discussion worldwide, especially concerning religious and legal considerations in countries such as Iran. According to the results of this qualitative study, a significant challenge is the frequency of sperm donations, which raises concerns about potential inbreeding among children conceived through these donations. Moreover, if donors are not screened for genetic or infectious diseases, there is a risk of transmitting such diseases to the resulting embryos. Currently, Iran lacks a national protocol governing donor requirements and limits sperm donation. For this reason, the experts participating in this study considered designing a protocol to be the highest priority vis-a-vis solving these challenges. While experiences from other countries in developing such protocols can be informative,^13^ any nation-tailored protocol must reflect its unique circumstances and needs, outlining donor criteria such as confidentiality, eligibility age, donation limits, and the rights of both sperm recipients and donors.

 Another important point emphasized by the experts was that to address concerns about disease transmission from donors to fetuses, consultation and screening tests are essential before donation. Therefore, the national protocol should delineate the required tests for donors and recipients, indicating which tests are mandatory or optional. Also, according to the experts, since discovering hidden diseases during screenings can cause emotional distress for donors, infertility centers should offer follow-up support and psychological counseling as part of their services and this important issue should also be included in the protocol. Moreover, considering the religious context of Iran, the national protocol must address jurisprudential and legal questions to assist donors and recipients with any related concerns and all infertility centers should operate based on the same protocols and procedures so that if a couple goes from one infertility center to another, there is no need to repeat previous examinations. Although a meta-analysis has not found a greater risk of birth defects, chromosomal abnormalities, or mental developmental disorders among children conceived through sperm donation during 10 years of follow-up,^14,15^ the experts of this study emphasized obtaining informed consent before treating infertile couples, allowing them to make informed decisions about their treatment and knowing the potential long-term complications and legal issues related to donation. In addition, existing studies tend to focus more on the ethical challenges of sperm donation than on disease transmission but some references have emphasized the importance of genetic, infectious, and psychological screenings before donation^16^ and the assessment of children for potential long-term complications in adulthood.^17^

 The study noted that deficiencies in laws governing the donation of embryos, sperm, and eggs, along with inadequate oversight of infertility centers, have commercialized the treatment process, turning it into a profitable business for brokers. Some couples are even seeking infertility treatments without necessity, including for sex selection of the fetus. Therefore, before the protocol is drafted, it is essential to identify and address these deficiencies in donation laws with the expertise of medical, ethical, jurisprudential, and legal professionals to avoid unresolved moral, religious, or legal challenges during the creation of the national protocol.

 Another practical suggestion that experts put forward in response to donation challenges in Iran was establishing a central registry for managing donor and recipient information and tracking activities at infertility centers. This registry can oversee sperm donations to prevent illegal activities and provide critical information for treatments, such as bone marrow transplants. According to experts, donors’ children may seek knowledge about their genetic origins and biological parents for reasons related to inheritance or emotional connections, and the registry can meet both their therapeutic and emotional needs. Therefore, legislation and national protocols should address inheritance issues, clarifying the rights of children born from donations regarding claims to their biological parents’ estates. The participants suggested leveraging the influence of senior state officials to reform donation laws and engage judicial research institutes in collaborative efforts.

 Based on our conclusion and interpretation of these statements, conducting qualitative research with managers, specialists, donors, and both current and former recipients of infertility centers can help identify and address many challenges related to donations, providing valuable insights for officials and researchers. As shown, the issue of donations can be influenced by various factors, such as the time and location of the research, and therefore, it is essential to conduct ongoing research in different cities across Iran and update national or regional guidelines as needed. In this regard, a study by Isaksson et al, which focused on couples receiving donations, highlighted the importance of understanding donor characteristics such as age, number of children, and medical history. The study emphasized the need for donor anonymity and the absence of a genetic parent-child relationship.^18^ In a study from Iran on recipients of donations, the principle of respecting recipients’ autonomy was underscored through comprehensive counseling, informed choice, and consent, which was identified as the most significant ethical challenge faced by infertility centers.^19^ Furthermore, a study in the United States involving children conceived through sperm donation revealed that many of them sought recognition of their biological donors. They desired a positive perspective from healthcare professionals regarding pregnancies resulting from donations and preferred not to have the donor’s information concealed, viewing their biological donors as their real parents.^20^ As the use of assisted reproductive methods continues to expand^21^, there is an increasing need for such studies in Iran, particularly qualitative studies, to better understand and address the emotional needs of both donors and recipients.

 Based on the opinions of the participants in this study and findings from other countries, donors typically donate due to financial necessity; however, moral and spiritual motivations may also be present.^22^ The results of this study indicate that one of the future concerns for donors is the question of their role as biological or third parents. In the study by Pennings, it was noted that sperm donors are subject to two harms: stigmatization and the concept of attributional fatherhood. The desire to avoid these harms leads many men to be reluctant to donate or to keep their identity and the details of their sperm donation confidential. Furthermore, as children resulting from donations often seek to identify their sperm donors and the parents of these children tend to prefer anonymity, sperm donation presents additional challenges.^23^ All these reported contradictions should be examined within the Iranian society, and the protocols surrounding sperm donation should be adjusted based on the findings of Iranian studies.

 In the context of egg donation, the participants highlighted the complications associated with ovarian stimulation treatments for the donor rather than focusing on moral dilemmas or the potential risk of passing genetic diseases to the donor’s child. While the risk of ovarian hyperstimulation syndrome is manageable and can be mitigated with appropriate precautions,^24^ particularly for healthy young women,^25^ the medical ethicist involved in this study referred to the unfortunate case of a young girl who died as a result of egg donation. This underscores the necessity of obtaining informed consent that thoroughly outlines all potential complications and emphasizes the importance of monitoring the donor’s condition during the stimulation of ovulation. Since ovaries play a crucial role in reproductive health, this issue must be addressed in national protocols. Additionally, egg donation may present psychological challenges for the donor, highlighting the need for psychological support and counseling throughout the process.^26^

 Since this study was conducted during a group discussion and only twelve experts could participate, this is one of the limitations of this study. Of course, the strength of this study is that key informants were invited to conduct interviews. However, since it was not possible to conduct individual interviews with them, due to their busy schedules, it is suggested that in future qualitative or mixed methods studies, individual face-to-face interviews with experts should be conducted.

 It is worth noting that to prevent selection bias, specialists from various hospitals and universities of medical sciences in Tehran were invited to the interviews. In addition, to prevent confirmation bias, during the interviews, the researcher asked the specialists to provide their opinions if they had any opinions confirming or rejecting the statements of other specialists. Since data analysis was performed by a reproductive health specialist who has no professional activity in the field of infertility treatment and approached the data analysis with an open mind and without prejudice, this also reduced the risk of confirmation bias.

## Conclusion

 Sperm donation raises several ethical and legal issues, including concerns about inbreeding, confidentiality, the financial expectations of biological parents, and the risk of disease transmission. In the case of egg donation, the health of donors is prioritized during the ovulation stimulation process.

 Ambiguities in laws related to sperm, egg, and embryo donation in Iran, as well as lack of a central registration system and a national protocol, can lead to parallel treatment in infertility centers and the possibility of financial abuse or unnecessary receipt of assisted reproductive services.

 Current research does not thoroughly address all relevant aspects and stakeholders involved in this matter. Therefore, it is crucial for policymakers and health researchers to carefully evaluate these situations and develop a comprehensive national protocol based on solid scientific evidence. The Ministry of Health should establish a central registry to ensure that the legal process of donation is upheld in both public and private centers, enabling infertile couples to pursue parenthood with fewer challenges and concerns.
